# Supplementary material for: Micro Light Flow Controller on a Programmable Waveguide Engine
Source: Micromachines (Basel). 2022 Nov 16;13(11):1990. doi: 10.3390/mi13111990 (PMC9699270; doi:10.3390/mi13111990)
Supplement: Supplementary file 1 [file micromachines-13-01990-s001.zip › Source Code S1 optimized program of the micro light flow controller.pdf]

Optimized program in micro light flow controller

```matlab
%#####################################################
% Micro Light Flow Controller on a Programmable Waveguide Engine
% Tao Chen 1,2,†, Zhangqi Dang 1,2,†, Zeyu Deng 2, Zhenming Ding 1,2, and Ziyang Zhang 2,*
% 1 College of Information Science and Electronic Engineering, Zhejiang University, Hangzhou 310027, China
% 2 Laboratory of Photonic Integration, School of Engineering, Westlake University, Hangzhou 310024, China
% * Correspondence: zhangziyang@westlake.edu.cn
% † These authors contributed equally to this work.
%#####################################################

clc;clear; % Clear screen and variable in workspacce.

% Create folder#######################################
png_path = [pwd,'\png\'];% Png data path from camera.
mkdir(png_path);
bar_fig_path =  [pwd,'\bar\']; % Bar fig path.
mkdir(bar_fig_path);
spot_path =   [pwd,'\spot\']; % Clipping light spot path.
mkdir(spot_path);
filename_number_intensity=[pwd,'\number_intensity.txt']; % Create the number intensity txt file.
fid=fopen(filename_number_intensity,'w+');
% Create folder#######################################

% Parameters setting###################################
background = imread([pwd,'\background.png']); % Read the background image.
pause_time = 0.5; % Set the pause time between the applied current and image capture from IR camera, unit: second.
total_sweep_times = 300; % the maximum sweeping times.
objective_sum = 1.02e7; % Set the total output power (pixel counts) of the original 1x2 switch
objective_state_percent = [0.33,0.33,0.33]; % The target percents of the 3 outputs.
% Parameters setup#####################################

% MCU-based current source setup ###################
% Set the switch sate of the channel in current source.[0: power off;1: power on]
m = modbus('serialrtu','COM3','Timeout',20); % Connect with the current source by serial cable through modbus protocol.
CH1=0;  % off
CH2=0;  % off
CH3=1;  % on, Electrode 3 in this work.
CH4=0;  % off
CH5=1;  % on, Electrode 4 in this work.
CH6=0;  % off
CH7=0;  % off
CH8=0;  % off
CH9=0;  % off
CH10=0; % off
CH11=0; % off
CH12=1; % on, Electrode 2 in this work.
CH13=0; % off
CH14=1; % on, Electrode 1 in this work.
CH15=0; % off
```

```
CH16=0; % off

CH_switch=[CH1,CH2,CH3,CH4,CH5,CH6,CH7,CH8,CH9,CH10,CH11,CH12,CH13,CH14,CH15,CH16]; %↙
Create the switch state vector.
write(m,'holdingregs',1,CH_switch); % Write the switch state vector into the MCU↙
register to make it effect.
% Write(port name of the register,'holdingregs',start_address,switch_state_vector)
% MCU-based current source setup ###################

% CCD camera initialization
try
imaqreset; % Image acquisition toolbox module initialization.
vid = videoinput('gige', 1, 'Mono16'); %  Connect the CCD camera through the ethernet↙
cable by GigE vision protocol.
src = getselectedsource(vid); % Get the camera.
src.PacketDelay=70000; % Set the packet delay.
src.PacketSize =1486; % Set the packet size.
imaqmex('feature', '-gigeDisablePacketResend', true); % Blank the Warnings.
preview(vid); % Preview the camera data.
catch
% If the camera has been activated, skip this process.
end

% The optimization process ########################
sweep_number = 0; % Set the initial sweep number.
data_current_config_all = []; % Create the current config matrix.
tic
while sweep_number <= total_sweep_times % start the optimization process
data_all =[];
initial_current = round(double(rand(1,16)*9000)); % Set the initial current↙
configuration, unit: μA
% initial_current = [0  0   x   0   x   0   0   0   0   0   0   x   0   x   0   0];
% or set the specifc current value

sweep_number = sweep_number +1; % Or set the sweeping number iteration.

if sweep_number == 1 % if sweep_number is 1, the output current is the initial↙
current.
    output_current = initial_current;
end

% Write the channel current config into the register.
write(m,'holdingregs',17,output_current);
% Write(port name of the register,'holdingregs',start_address,output_current_vector).

% if sweep_number > 1
% Read the switch state and current config in the register and save on the disk.
data_read = read(m,'holdingregs',1,32);

pause (pause_time) % The extra pause time for the data process and thermal↙
equilibrium.

try %
img=getsnapshot (vid); % Capture the image in the CCD.
name_png = [png_path,num2str(sweep_number),'.png']; % Set the serial number of the png↙
```

```
document.
imwrite(img,name_png); % Save the image in png format.

catch % Self-recovery for the CCD camera if the CCD is disconnected or crash
% Reinitialize the IR camera ##############
imaqreset; % Reset imaqtools
vid = videoinput('gige', 1, 'Mono16');
src = getselectedsource(vid);
src.PacketDelay=PacketDelay;
src.PacketSize = 1486;
preview(vid)
% Reinitialize the IR camera ##############

img=getsnapshot (vid); % Capture the image in the CCD
name_png = [png_path,num2str(sweep_number),'.png']; % Set the serial number of the png↙
document.
imwrite(img,name_png); % Save the image in png format.
end

%***************************************************Captured image process
% Parameters setting.
% Set the middle x and y axis of the 3 outputs, which is recognized in advance.
x_mid = [306,304,302];
y_mid = [134,322,511];
% Set the size of the spot.
size_x = 40;
size_y = 40;

start = img; % Read the captured image.
img_process = start - background; % The process image data is removed the background↙
information.

% The following process is to clip the light spot from the captured image.
location = 0; % subplot number
figure(1)
port_intensity_data = []; % Save the calcuated power information of the 3 output↙
ports.

for q=1:1:3 % q is the output port number.
    x_min = floor(x_mid (1,q) - size_x/2);
    x_max = floor(x_mid (1,q) + size_x/2);
    y_min = floor(y_mid (1,q) - size_y/2);
    y_max = floor(y_mid (1,q) + size_y/2);
    port_region = img_process (x_min:x_max,y_min:y_max); % Save the cropped light spot↙
data.
    intensity_port = sum (sum (port_region)); % The intensity information by summing↙
up the pixel count.
    port_intensity_data = [port_intensity_data; q intensity_port]; % Save the↙
intensity data.
    location = location+1;
    % Plot the subplot figure of the information.
    subplot(1,3,location)
    imshow(port_region);
    caxis([0,20000])
end
```

```
% Save the cropped spot figure.
spot_name = [spot_path,num2str(sweep_number),'_spot.png'];
saveas(figure(2),spot_name);

% Convert the output intensity into the percent of objective sum.
port_only_intensity_data = port_intensity_data(:,2)';
port_only_intensity_data_percent = port_only_intensity_data/objective_sum;

%**************************************************

% Write the parameter data into txt document.
data_all = [sweep_number, data_read, port_only_intensity_data↙
port_only_intensity_data_percent];
[m_data,n_data] = size (data_all);
for print_number = 1:1:n_data+1
if print_number <=n_data
fprintf(fid,'%f ' , data_all(1,print_number));
end
if print_number ==n_data+1
fprintf(fid,'\n ');
end
end

% Real-Time judgement.
actual_state = port_intensity_data(:,2)'; % The absolute value of the output lights.
actual_state_percent = port_only_intensity_data_percent; % The percent value of the↙
output lights.

% Caculate the Cost Function (CF).
diff_1 = 1-abs(objective_state_percent(1)-actual_state_percent(1))/↙
(objective_state_percent(1)+actual_state_percent(1));
diff_2 = 1-abs(objective_state_percent(2)-actual_state_percent(2))/↙
(objective_state_percent(2)+actual_state_percent(2));
diff_3 = 1-abs(objective_state_percent(3)-actual_state_percent(3))/↙
(objective_state_percent(3)+actual_state_percent(3));
sum_actual_state_percent = sum (actual_state_percent);
sum_objective_state_percent = sum (objective_state_percent);
cost_function = objective_state_percent(1)/sum_objective_state_percent*diff_1+...
    objective_state_percent(2)/sum_objective_state_percent*diff_2+...
    objective_state_percent(3)/sum_objective_state_percent*diff_3;

% Establish the data library for the following enquiry.
data_current_config_all = [data_current_config_all;sweep_number output_current↙
cost_function];

if sweep_number == 1 % If the sweep_number == 1, the output_current is the best↙
current config.
    best_current_config = output_current;
    rand_NP_1 = 2*rand(1,16)-1; % Random fucntion.
    output_current = best_current_config + round(double(rand_NP_1*300)); % Adjustment↙
level 2.
end % if sweep_number == 1

if sweep_number > 1 %If the sweep_number > 1, compare the cost function.
```

```
    last_cost_function = data_current_config_all(sweep_number-1, 18);

    if   sum_objective_state_percent*0.80 <= sum_actual_state_percent ...
            &&  sum_actual_state_percent <= sum_objective_state_percent*1.20 ...% Only↙
the sum actual intensity is in the setting ranging.
            && cost_function >= 0.8 % and the cost funciton is >= 0.8, the minor↙
adjusting is applying.

    if  (cost_function - last_cost_function) <= 0.02 % Selective retention.

    best_current_config = data_current_config_all(sweep_number-1,2:17);
    output_current = round(best_current_config+ round(double(rand_NP_1*300)));
    else
    best_current_config = data_current_config_all(sweep_number,2:17);
    output_current = round(best_current_config+ round(double(rand_NP_1*300)));
    end % minor adjusting end

    else % the sum actual intensity is not in the setting ranging.
    output_current = round(double(rand(1,16)*9000)); % producing the new random↙
current.
    end % if sum_actual_state_percent >= 0.91

end % if sweep number > 1

% if the cost_function is >= 0.9, the optimization is completed.
if cost_function >= 0.9
    break
end

end % while end

data_all_proces = load ('number_intensity.txt'); % Save data.
xlswrite('number_intensity.xlsx',data_all_proces); % Save data.
write(m,'holdingregs',1,zeros(1,32)); % Clear all the registers.
toc
```
